# T-helper cells flexibility: the possibility of reprogramming T cells fate

**DOI:** 10.3389/fimmu.2023.1284178

**Published:** 2023-11-01

**Authors:** Julia N. Khantakova, Sergey V. Sennikov

**Affiliations:** Department of Molecular Immunology, Federal State Budgetary Scientific Institution “Research Institute of Fundamental and Clinical Immunology” (RIFCI), Novosibirsk, Russia

**Keywords:** CD4 T cells, Th17, Treg, plasticity, differentiation, reprogramming

## Abstract

Various disciplines cooperate to find novel approaches to cure impaired body functions by repairing, replacing, or regenerating cells, tissues, or organs. The possibility that a stable differentiated cell can reprogram itself opens the door to new therapeutic strategies against a multitude of diseases caused by the loss or dysfunction of essential, irreparable, and specific cells. One approach to cell therapy is to induce reprogramming of adult cells into other functionally active cells. Understanding the factors that cause or contribute to T cell plasticity is not only of clinical importance but also expands the knowledge of the factors that induce cells to differentiate and improves the understanding of normal developmental biology. The present review focuses on the advances in the conversion of peripheral CD4+ T cells, the conditions of their reprogramming, and the methods proposed to control such cell differentiation.

## Introduce

It has long been believed that mature T-helper (Th) lymphocytes are terminally differentiated cells of functionally distinct subpopulations that differ in transcriptional and cytokine profiles. However, recent studies have shown that T cells have varying degrees of plasticity, allowing them to adapt to specific challenges and acquire new characteristics and functions in immune responses, depending on the tissue microenvironment. Surprisingly, lymphocytes recognized as terminally differentiated Th1 and Th2 can change their functional specialization under certain microenvironmental conditions. Foxp3-expressing regulatory T cells (Tregs) was previously thought to be remarkably stable under both basal and inflammatory conditions (1, 2). But several recent studies have shown that changes in expression and stability FoxP3 during inflammation can alter the phenotypic properties of Tregs, converting Tregs effector function, thus confirming a certain degree of plasticity cells (3–7). T-helper 17 cells (Th17) are largely unstable and can reprogram into many types of T-helper lymphocytes. Determining the possible plasticity of T cell subpopulations in various tissues is highly relevant for future therapeutic interventions in such diverse immune pathologies as chronic viral infections, cancer, and autoimmune diseases. In this review the authors will not discuss the processes occurring in the thymus during positive and negative selection, and stability and flexibility CD8+ cytotoxic T cells as they are widely analyzed in other publications (8–11). The present review focuses on the advances in the conversion of peripheral CD4+ T cells, the conditions of their reprogramming, and the methods proposed to control such cell differentiation.

## The differentiation of T-helper lymphocyte subpopulations in the periphery.

T cells differ from most somatic cells due to continued differentiation in adulthood depending on the encountered antigen, as well as the ability to reprogram between different CD4+ T-helper cell lineages (12–18). CD4+ T-lymphocytes has the great potential to perceive various activating signals such as cytokines, chemokines, and other environmental factors, in response to which cascades of effector programs are triggered. Traditionally, the activation process of naïve T lymphocytes begins after 3 subsequent signals (19, 20): 1. T-cell receptors (TCRs) recognize antigens presented on the surface of cells in a complex with MHC. 2. Interaction of costimulatory molecules (CD4/CD8/CD28, etc.). 3. Creation of a cytokine microenvironment. Depending on the type of signal, the naive CD4+ T cell starts to express various transcription factors leading to the activation of transcription and translation of cytokine and chemokine genes necessary for eliminating specific pathogens or preventing immune-mediated pathologies (see **Table 1**).

**Table 1:** Role of signaling pathways and transcription factors in the differentiation of T lymphocyte subpopulations.

Pathway	Role	References
Ras-ERK1/2-AP-1	early determination of the CD8^+^ T cell memory	(21)
	proliferation and Th1 differentiation *in vitro;* inhibition of regulatory T cell (Treg) differentiation	(22)
	BATF regulate the differentiation of T helper (Th)17 cells and the conversion of CD4^+^Foxp3^+^ cells to CD4^+^IL-17^+^ cells.	(23)
	JunB activates the expression of Th17 lineage-specifying genes and coordinately represses genes controlling Th1 and regulatory T-cell fate.	(24)
IP _3_ -Ca ^2+^ -NFAT	Ca^2+^ signals control proliferation, differentiation, apoptosis, and a variety of transcriptional programs	(25–27)
	development and function of regulatory T cells	(28)
PKCθ-IKK-NF-κB	PKCθ is recruited to the immunologic synapse between APC and T cell, triggering T cell activation activation and proliferation of mature B and T cells	(29–34)
	cytoskeletal polarization in T cells	(35)
	PDK1 is essential for integrating the TCR and CD28 signals and activating NF-κB and PKCθ in T-cells.	(36–39)
TSC1/2-mTOR	mTOR is involved in the activation, differentiation, and function of effector T cells (Th1, Th2, Th17), but blocks Treg cell formation	(40–42)
Phosphatases	Phosphorylation and dephosphorylation of TCR signaling molecules affect the formation of signaling complexes and the propagation of TCR signals	(43–45)
	SHP-1 negatively regulates the differentiation process from naïve T cells to Th1 or Th2 effector T cells and/or the proliferation of differentiated T cells	(46)
	SHP2 is to suppress the differentiation of T cells to the Th2 phenotype.	(47)
	Forced expression of miR-181a enhances the TCR response in mature T cells, making activation by antagonistic ligands possible	(48)
	Suppression of *Ptpn22* (SHP2 gene) increases the expansion and function of effector/memory T cells	(49)
	Recruitment of the inhibitor phosphatases PP2A and SHP2 is involved in the induction of partial anergy of T _reg_ cells in response to TCR and CD28 stimulation	(50)
	DUSP2 inhibits signaling through STAT3 and restricts Th17 differentiation	(51)
	PTPN2 may support memory CD4+ T-cell responses by shaping memory effector functions or prolonging lymphocyte survival acting on STAT1	(52)
Ubiquitination and degradation	Roquin1/2 ligases maintain immune tolerance and block differentiation of effector and Tfh cells	(53)
	LMP7 (part of the immunoproteasome) promotes Th1 and Th17 differentiation, has no effect on Th2 cells, and blocks Treg cells	(54)
	overexpression of Stub1 in Treg cells abrogated their ability to suppress inflammatory immune responses in vitro and in vivo and conferred a T1-like phenotype by promoting degradation of the Foxp3	(55)
	E3 ligases, including Cbl-b, the gene related to anergy in lymphocytes	(56)
DAG kinases	loss of DGKα and/or DGKζ leading to hyperactivation, impaired induction of anergy	(57, 58)
	loss of DGKζ enhanced TCR signaling, and increased generation of nT_reg_ cells in mice.	(59)
**Ikaros (including** Helios and Aiolos)	In mice, depending on the mutation in Ikaros, the number of T, B, and NK lymphocytes and their early precursors are absent or significantly reduced, but impaired hyperproliferation and differentiation of CD4 lymphocytes may be observed	(60, 61)
**GATA-3**	Th2 cell differentiation, deletion of Gata-3 in early or late stage thymocytes showed the arrest of the DN3 population with decreased DN4, DP, and SP populations or impaired differentiation into CD4+ T cells, respectively	(62–64)
	*Gata-3* induces expression of the *Zbtb7b* gene, encoding a ThPOK transcription factor that inhibits differentiation of DP-thymocytes into CD8-SP cells and promotes differentiation into CD4-SP cells	(65)
**Notch**	Provides differentiation of common lymphoid precursors into T-lymphocytes. In the absence of Notch, B-lymphocytes are formed	(66, 67)
STAT (signaling transducer and activator of transcription)		
**STAT1**	STAT1 Is Required for IL-6–Mediated Bcl6 Induction Tfh	(68)
	Inhibits differentiation of Th17 lymphocytes	(69)
	Together with Tbet, it is a key factor in Th1 lineage differentiation, IFNγ-STAT1-T-bet pathway	(70, 71)
**STAT3**	Required for RORγt induction and Th17 lymphocyte differentiation	(72)
**STAT4**	IL-12 activates STAT4, which is critical for Th1 lymphocytes. Stat4-deficient lymphocytes differentiate predominantly into Th2 lymphocytes	(73, 74)
	STAT4 is important for activating the function of IL-23-stimulated Th17 pathogenic lymphocytes	(75)
**STAT5**	Activation of STAT5 by IL-2 stimulates differentiation of Th1, Th2, and Th9 cells but suppresses the development of Th17 and Tfh cells	(76–82)
	Regulates FoxP3 expression and Treg differentiation	(83)
**STAT6**	Activation of STAT6 under the influence of IL-4 triggers Th2 lymphocyte differentiation	(84–86)
	STAT6 suppresses FoxP3 expression and differentiation of iTreg cells	(87, 88)
**Blimp1**	Blimp-1 suppresses Tfh cell differentiation through suppression of Bcl-6	(89, 90)
**Bcl-6**	Tfh cell differentiation, master regulator	(90–92)
**Bcl-3**	Suppresses Th9 differentiation through limiting glutathione availability	(93)
**BATF**	Binds to the *Il6* promoter and enhances IL-6 cytokine secretion	(94)
	Together with STAT3, it is required for RORγt induction and Th17 lymphocyte differentiation	(95)

TCR signaling plays a critical role in the selection of differentiation lineages of various CD4+ T-cell subpopulations. The T-cell receptor TCR belongs to the family of immunoreceptors. It consists of 2 chains: α/β or γ/δ. In this review, α/β TCRs will be described, and all further references to “TCR” will refer specifically to the αβ TCR. We will not discuss molecular models of TCR signaling initiation, which leads to different cellular responses, as they are widely analyzed in other publications (96–98). A brief description of intracellular events that occur during the interaction of TCR and pMHC is described in **Box 1**. The table summarizes some data on the role of different signaling pathways and transcription factors, in the differentiation of T lymphocyte subpopulations [adapted and added from 99)].

In doing so, the affinity strength of antigens to TCRs was shown to be sufficient to induce differences in the physiology of differentiated T cells. When naïve CD4+ T cells are subjected to strong TCR stimulation, differentiation of Th1 preferentially proceeds, both *in vitro* and *in vivo* (100–102). Conversely, weak TCR signaling favors Th2 cell differentiation (101, 103). Besides, the strength of TCRs signaling influence on initial cytokine production: low antigen concentrations trigger interleukin (IL)-4-independent IL-4 production during the first 24 hours after T cell engagement, whereas stimulation with high concentrations suppresses early IL-4 production but enhances interferon production (IFN)-γ (19). The issue of whether differences in the strength of TCRs signaling affect Th17 cell differentiation remains controversial (104).

Box 1 **Intracellular events TCRs initiation.**

The TCR consists of an extracellular region, a transmembrane region, and a shorter cytoplasmic tail. At the same time, none of the TCR chains has internal kinase activity or the ability to interact with non-receptor tyrosine kinases (Mariuzza et al., 2020). The TCR forms complexes with δ-, γ-, ε- and ζ-chains of CD3, essential for cell surface expression and intracellular signaling. The interaction between the extracellular domains of CD3 immunoglobulin subunits and TCR chains is necessary for the formation of the antigen-recognition complex of T cells. Despite the presence of longer cytoplasmic fragments, CD3 chains also lack the enzymatic activity that would support intracellular signal transduction during antigen recognition. For this purpose, co-receptor CD4 are located near the TCR chains, which bind the TCR to the Src family kinases Lck and Fyn, which engage and phosphorylate the CD3 immunoreceptor tyrosine-based activation motif (ITAM) complex and initiate the downstream signaling cascade, ultimately leading to T cell survival, differentiation, and effector functions (Walk et al., 1998). The combinatorial mutation of ITAM TCR demonstrated their central importance for T cell development and function (Bettini et al., 2017; Holst et al., 2008; Love & Hayes, 2010). Decreased ITAMs lead to increased Treg formation in mice (M. O. Li & Rudensky, 2016). Phosphorylation of ITAMs enables recruitment of the TCR-associated protein Zeta-chain-associated protein kinase 70 (Zap70), which is then phosphorylated by Lck. Zap70 then phosphorylates four key sites on the linker for activation of T cells (LAT), which allows the proteins to be recruited to the LAT signalosome. The subsequent effect is the activation of the Rat sarcoma (Ras)/extracellular signal-related kinase (Erk)/Activator protein 1 (AP-1) pathway, Protein kinase C-θ (PKCθ)/κB kinase (IKK)/nuclear factor-κB (Nf-κB) pathway, and the calcium-dependent Calcineurin/nuclear factor of activated T cells (NFAT) pathway (Hwang et al., 2020; Shah et al., 2021). Transcription factors downstream of these pathways, NFAT, Nf-κB, and AP-1 contribute to IL-2 transcription as well as to IL-2RA transcription, which encodes the α-chain of the IL-2 receptor (CD25) (Y. Li et al., 2022). Phosphorylation and dephosphorylation of TCR signaling molecules such as Syk and ZAP-70, as well as ubiquitination and degradation of CD3ζ, PKCθ, ZAP-70, phospholipase C-γ1, and phosphoinositide-3-kinase, negatively regulate TCR signaling pathways. Signal transducer and activator of transcription (STAT) proteins control clone-specific transcription factor expression and also control epigenetic changes, such as histone modifications or DNA methylation, which open specific DNA sites for transcription.

It should be noted that the strength of TCR signaling also regulates the differentiation of regulatory T cells (Treg) (105, 106). A low density of high-affinity ligands is important for the stable induction of peripheral Treg cells (107, 108). Longer TCR-pMHC residence time as well as high-affinity TCRs are positively associated with follicular helper T cell (Tfh) differentiation (102, 109). Stable Th 9 function requires sustained TCR signaling and the IL-9 secretion (110, 111).

**Figure 1:**
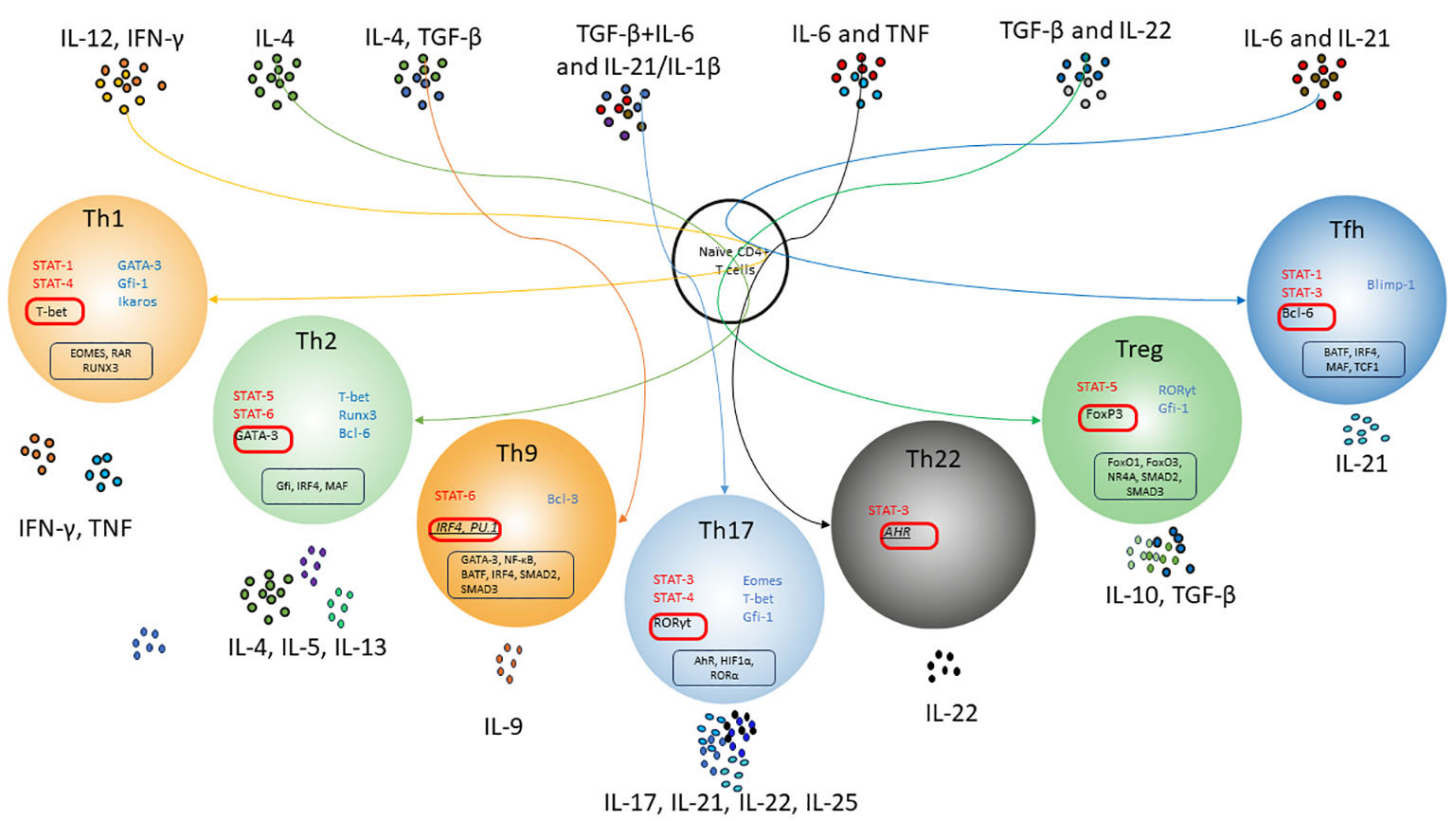
Scheme of differentiation pathways of naïve CD4+ T-lymphocytes depending on the cytokine microenvironment. T cell receptor (TCR) signals and polarizing cytokine signals activate and/or enhance special transcription factors, which allow naïve CD4 T cells to differentiate into subsets of T helper cells. Activation of IL-4 and Stat6 enhances GATA-3 expression and T helper 2 (Th2) cell differentiation, which strongly suppresses the expression of Th1-related genes. Under the influence of IL-12 and IFN-γ, Tbx1 together with STAT4 induces optimal production of IFN-γ and triggers the differentiation of Th1 lymphocytes. That's also suppresses Th2-related genes and IL-17A expression. With the synergistic influence of IL-6, IL-21 and TGF-β, Th17 differentiation occurs due to STAT3 activation and induction of RORγt expression. TGF-β may promote Treg cell differentiation by influencing FoxP3; moreover, conventional T helper cells are given a regulatory phenotype with enhanced Foxp3 expression. Th17-associated factors suppress Foxp3 expression through ROR-γt binding or STAT3 signaling. Tfh cell differentiation occurs upon activation of STAT3, presumably by IL-6 and IL-21, which are triggered by the transcriptional regulator Bcl-6. Bcl-6 represses alternative T helper cytokines such as IFN-γ and IL-17A. Th9 lymphocytes are formed under the combined influence of IL-4 and TGF-β due to increased expression of the transcription factor PU.1 and production of IL-9. AhR has been considered a transcription factor for IL-22 produced by Th22 cells Red color indicates activating transcription factors. Blue color indicates inhibition transcription factors. Master regulators are shown in red boxes. Suggestive master regulators for Th9 and Th22 are indicated italic. Co-factors of master regulators are shown in black boxes.

An important principle of CD4+ T-cell differentiation is that one of the characteristic cytokines produced by each differentiated cell also plays a critical role in the induction of such cells, potentially providing a powerful positive feedback loop (**Figure 1**). These “feedback” cytokines are IFN-γ for Th1, IL-4 for Th2, IL-21 for Th17, and TGF-β for iTreg. Thus, exposure to IL-12 or IFN-γ activates STAT-1 and triggers the expression of the transcription factor T-bet, which is required for Th1 lymphocyte differentiation (112). IL-4 signals activate STAT6 and further Gata3 required for polarization toward Th2 lymphocytes (J. 113). However, if TGF-β is additionally present in the medium, other transcription factors, PU.1 and IRF4, are activated and direct differentiation toward Th9 lymphocytes (114). A combination of TGF-β and IL-6 or IL-21, via the STAT3 pathway (115), induces the expression of RORγt and directs CD4+ differentiation toward Th17 (116, 117). Besides, TGF-β is essential for Treg cell differentiation through its effect on the FoxP3 transcription factor (118). In the absence of TGF-β, IL-6 and IL-21 also induce the expression of another transcription factor, Bcl6, the major transcription factor of Tfh cells, via STAT3 (119). STAT3, stimulated by IL-6 and TNF, is required for the differentiation of Th22 cells expressing AhR as their major transcription factor (120).

Thus, CD4+ T-cell plasticity is influenced by a number of external (e.g., cytokines, various metabolites) and internal factors. Effector CD4+ T-lymphocytes depending on the cytokine microenvironment can develop into different subpopulations of cells with specific functions. It is worth noting that a meta-analysis of genes previously associated with Th1, Th2, Th17, and Treg populations showed that except for master regulator genes (T-bet, GATA-3, Bcl-6, FoxP3 and RORγt), all other genes considered specific showed significant variability (121). This confirms the ability of previously polarized T cells to change their phenotype and repolarize toward other differentiation lineages.

## Possibilities of reprogramming CD4+ lymphocyte subpopulations

For a long time, it was believed that different subpopulations of CD4+ T cells were terminal lines of differentiation of T-helper cells. However, accumulating data show the plasticity of CD4+ T cells and their ability to acquire different properties and functions (122, 123). T-helper lymphocytes can express not only their clonally specific cytokines and Transcription factors but also simultaneously markers of other T helper lineages, making them similar to hybrid cells (124). This is especially shown for Th17-Treg pairs (125, 126) and Th17-Th1 cells (127, 128). Still, there is also evidence for reciprocal reprogramming of other Th lines, which will be discussed further.

### Mutual plasticity of Th1-Th2 lymphocytes

The earliest discovered and most studied subpopulations of helper T lymphocytes are Th1 and Th2 cells. Th1 and Th2 subsets seem to be the most stable, as they are regulated by mutually repressive and self-amplifying transcription and signaling factors (T-bet and IFN-γ for Th1 and GATA-3 and IL-4 for Th2) (19, 100–103). However, it was soon shown that forced expression of the GATA3 regulator GATA3 in Th1-lymphocytes using the viral vector Th2-master induced the production of Th2-cytokines (IL-4 and IL-5), as well as CCR4. At the same time, transduced cells partially preserve their Th1-specific profile (expression of IFN-γ and IL-12Rβ2) (129). Conversely, expression of T-bet (Tbx21) in Th2 cells promoted a Th1 phenotype. It was also shown that memory CD4+ T cells under repeated stimulation *in vivo* were able to produce cytokines of the oppositional lineage, indicating the existing functional plasticity in T helper cell responses (130). However, this flexibility decreases as CD4+ lymphocytes mature (131), which, in general, is characteristic of the differentiation dynamics of any multipotent progenitor cells. This is thought to occur due to chromatin remodeling in cytokine gene loci to increase the efficiency of effector cytokine production and inhibition of opposing cytokine programs (132).

Chromatin modification is responsible for the suppression of the differentiation program of oppositional Th cell lines. Thus, T-bet activation in Th1 causes loss of HDAC-Sin3A at the *Ifng* locus and promotes IFN-γ expression (133). In Th2 cells, IFN-g production is suppressed due to the deposition of the repressive histone mark H3K27me3 to the *Ifng* locus (134). Demethylase Jmjd3 changes in histone methylation (H3K27me3 and H3K4me3 levels) in target genes and regulates Th1, Th2, and Th17 lymphocyte differentiation. Deletion of Jmjd3 results in Th2 and Th17 differentiation and blocks Th1 differentiation (135). After activation and differentiation of human Th2 cells, the permissive marks H3K9 acetylation and H3K4me3 were increased in the respective loci of the Th2-specific genes *Il-4, Il-5, and Il-13* (77).

Experimental studies showed that differentiated Th1 cells under the influence of IL-4 or helminths can be converted into an IFN-γ-producing Th2 lineage (136, 137). Conversely, treatment of Th2 lymphocytes with IL-12 and anti-IL-4 induces repolarization toward Th1 cells (138, 139). However, it is possible that reprogramming of the Th1 phenotype to the Th2 phenotype may reflect the growth of the Uncommitted Precursors population rather than the growth of the Th subpopulation (138). Another study shows that infections of Th2 lymphocytes with lymphocytic choriomeningitis virus, known for its strong induction of type I and type II interferons, results in the reprogramming toward GATA-3 + T-bet + Th1-lymphocytes capable of producing Th1 and Th2 cytokines (140). It was also shown that deletion of the IL-10 gene in naïve CD4+ lymphocytes led to Th1 lymphocyte differentiation even under Th2 differentiation conditions (141).

As mentioned previously, TCR and costimulatory signals can also influence plasticity between CD4+ T-cell subsets. TCR stimulation is ubiquitously required for cytokines to reprogram T cell subpopulations (140). The weaker stimulation of TCRs during priming *in vivo* makes possible significant plasticity in their cytokine production upon reactivation (142). Depending on signal strength and recall, the antigen dose also redirects subsets of T helper cells: higher antigen concentration promotes Th2 cell phenotypes in memory Th1 cells (143). At low antigen concentrations, Th2-lymphocytes differentiate with the participation of Itk kinase. Cells deprived of Itk kinase, even under conditions of stimulation with low levels of antigen, show increased expression of T-bet and differentiate into Th1 (144).

### Plasticity of Th1/Th2 to other Th lymphocytes

There are published data on mutual transitions of Th9, Tfh, and Th22 into Th1/Th2 lymphocytes. The flexibility of all the above populations with Th17 cells will be considered separately due to the great plasticity of Th17 lymphocytes.

Treatment of Th2 lymphocytes with TGF-β causes the loss of their characteristic profile and induces the secretion of IL-9, and in combination with IL-4, it drives the differentiation of Th9 cells (145). On the other hand, under Th1 culture conditions (in the presence of IL-12), Th9 lymphocytes can acquire a Th1 phenotype and produce IFN-γ *in vivo* (146, 147). Polyamines play a critical role in the regulation of Th2/Th9 balance. Endogenously generated polyamines enhance GATA-3 expression and promote Th2 cell differentiation (148). Under polyamine deficiency, even under Th2 environmental conditions, expression of Th9-related genes (*Il9, Irf4, and Batf3*) is enhanced through suppression of GATA-3.

Helminth load results in reprogramming of Th2 lymphocytes into the Tfh population, via the Smad3/Smad4 and IRF-4 activation pathway (149, 150), and expresses the canonical Tfh markers Bcl6 and IL-21, as well as GATA3, a master regulator of Th2 cell differentiation (151). On the other hand, the formation of Th2 lymphocytes from IL-4-producing Tfh lymphocytes was shown in allergic asthma *in vivo*. Impairment of Tfh cell responses during the sensitization phase or Tfh cell depletion prevented Th2 cell-mediated responses following the challenge (152). In addition, pre-differentiated Tfh cells in Th1-, Th2-, or Th17-conditions acquire the ability to produce IFN-γ, IL-4, or IL-17, respectively, while retaining their Tfh potential (capacity to produce IL-21) (153).

Tfh can also be reprogrammed toward Tr1 cells *in vivo* upon Blimp1 upregulation (154). It was also shown that deletion of the IL-6 gene in naïve CD4+ lymphocytes led to Tr1 differentiation even under Th2 differentiation conditions (141). Th1 lymphocytes under IL-12 hyperstimulation and under conditions of high TCR ligation/chronic infection can also switch from proinflammatory effector cells to Tr1 cells producing IL-10 (155–157). It is important to note that IL-10 production by CD4+FoxP3- T-lymphocytes is considered to be the main marker of the Tr1 cell population. To date, data on cytokine production by different subpopulations of Th cells (Th1, Th2, and Th17) were obtained. Such IL-10+Th cells can control their own effector functions by turning IL-10 production on and off. Thus, Tr1 cells can be very heterogeneous and do not represent a separate lineage.

Taken together, the present data demonstrate the existing flexibility of Th1 and Th2, capable of acquiring different functional and phenotypic properties upon repeated antigen stimulation and under the influence of the respective microenvironment.

### Mutual plasticity of Treg and Th17 lymphocytes

Th17 and peripheral Treg differentiation are closely linked and are likely necessary to maintain tolerance and prevent the development of inflammatory diseases (158). Data from fate-mapping experiments in mouse models identified transdifferentiation events of Th17-to-T regulatory cells (12). In humans with autoimmune diseases, an increase in the number of IL-17-producing FoxP3+ Treg cells in peripheral blood correlates with disease severity (4, 159–162). A better understanding of the mechanisms underlying Th17-Treg transdifferentiation in the human condition may be critical for resolving inflammation in autoimmune diseases.

Upon stimulation with TGF-β, naive CD4+ T cells undergo a dual expression stage of the transcription factors Foxp3 and RORγT (163–165) and, depending on the microenvironmental factors, differentiate into Treg or Th17 cells. High levels of TGF-β, retinoic acid, and IL-2 activate Foxp3 and support the differentiation of Treg cells (6). Combination of TGF-β and IL-6 or IL-21, via the pyruvate kinase M2 and STAT3 pathway (115), induces the expression of RORγt and enhances CD4+ differentiation toward Th17 (116, 117, 166, 167), by inhibiting the expression of Foxp3 (X. 168). Decreased STAT3 activation upon treatment of T cells with CK2 inhibitors results in decreased expression of the IL-23 receptor, required for optimal differentiation and maintenance of Th17, and increased differentiation of FoxP3+ Treg cells (169, 170). Enhancement of aryl hydrocarbon receptor (AHR) expression under the influence of TGF-β promotes phosphorylation of SMAD2/3 and STAT3, enhances Th17 lymphocyte differentiation, and blocks Th1 phenotype cell differentiation (IL-2 and T-bet) (167). On the other hand, AhR promotes transactivation of *Il10*, and could potentially reprogram inflammatory Th17 lymphocytes toward IL-10+ Tr1 and termination of the immune response (12). In addition, the master regulators themselves are in reciprocal interactions: RORγT interacts directly with exon 2 of the Foxp3 gene to suppress development and activate Th17 cytokine transcription. Similarly, Foxp3 can bind RORγT to suppress IL-17 production (168).

The Tec family kinase, Itk, plays an important role in Treg/Th17 differentiation (144, 171, 172). Itk deletion, even in a Th17-conditioned microenvironment, decreases Th17 lymphocyte differentiation but increases expression (171, 173). In addition, Itk -/- CD4 T cells gave an increase in the number of Treg cells when cultured under inducible Treg conditions. This was associated with decreased activation of the Akt/mammalian target of the rapamycin (mTOR) pathway and increased sensitivity to IL-2 (171). mTOR and HIF-1 are well-known integrators of metabolic signals responsible for initiating an adaptive cellular response, and in particular promote the development of Th17, but inhibit Treg differentiation. Optimal activation of mTOR leads to increased glycolysis and STAT signaling, promotes the development of Th17, while inhibiting the Treg differentiation (42). Constitutive activation of mTORC1 in mouse T cells by deletion of the mTORC1 tuberous sclerosis 1 (TSC1) gene-negative regulator enhances Th1 and Th17 cell differentiation and blocked Tregs differentiation (174). mTOR, in turn, stimulates HIF-1 to support glycolysis and is required to control the Th17 phenotype (175). Inhibition of mTOR can lead to increased fatty acid oxidation (FAO) and promote Treg differentiation (176).

High-NaCl conditions regulate the expression of FoxP3 and IL-17A through the p38/MAPK pathway involving NFAT5 and SGK1 and promote the differentiation of a stable, pathogen-specific, anti-inflammatory Th17 cell in human T cells *in vitro* in the presence of TGF-b (177). Butyrate promotes IL-10 Treg production (178–181) and promoted IFN-γ production under Th1 conditions, but not Th17 lymphocytes *in vitro* (182, 183). Inhibition of histone deacetylases (HDACs) in T cells by butyrate increased acetylation of p70 S6 kinase and phosphorylation of rS6, regulating the mTOR pathway required for Th17, Th1, and IL-10(+) Treg cell formation in the respective cytokine microenvironment (181). Interestingly, under butyrate exposure, IL-10 production increases in any CD4+ T-cell lineage due to the expression of B-lymphocyte-induced maturation protein 1 (Blimp1) (182). In addition, butyrate enhances histone acetylation at the Foxp3 promoter, promoting stable Foxp3 expression (178, 179). Neutralization of RAGE by the soluble receptor for advanced glycation end products (sRAGE) inhibits fatty acid synthesis and promotes polarization of CD4+ T cells toward Treg rather than Th17 (184).

*In vitro* and *in vivo*, biotin deficiency decreased FoxP3 expression and the number of Treg cell, but increased the expression of T-bet and RORγt and differentiation of CD4+ T cells into Th1 and Th17 cells with a significant increase in the production of proinflammatory cytokines IFN-γ, TNF, and IL-17 (185). Culturing in the presence of lactate reprograms Th17 lymphocytes toward Treg cells, significantly reducing IL-17A production and increasing FoxP3 expression through ROS-driven IL-2 secretion (186).

It is worth noting that co-expression of FoxP3 and master regulators of other Th cells was shown for peripheral FoxP3+ Treg. Thus, in the intestine, up to 40% of Treg cells simultaneously express FoxP3 and RORγT and show enhanced immunosuppressive function, effectively restraining intestinal inflammation (5). RORγT is thought to maintain stable FoxP3 expression in Treg cells by blocking T-bet activation, which prevents Treg from reprogramming into a Th1-like effector phenotype. Treg in B-follicles of secondary lymphoid organs similar to Tfh express Bcl6, CXCR5, and PD-1 (7). Other Treg, similar to Th1 lymphocytes, express T-bet and CXCR3 (187) and may originate from Foxp3+ T cells under the influence of IFN-γ and STAT1 (187), and may also develop from T-bet+CD4+Th1 cells through the action of the immunosuppressive cytokine TGFβ (3). A high frequency of such T-bet+FoxP3+ Treg cells is observed in oncology and potently inhibits T helper 1 (Th1) cell responses (188). Similar to Th2 lymphocytes, Treg located in barrier sites, including the gastrointestinal tract and skin, can co-express GATA3, CCR8, and ST2 (IL-33 receptor α-chain) (189). These double-positive Treg, unlike their Th counterparts, are unable to produce proinflammatory cytokines. It was suggested that by expressing these Th cell-associated molecules, they accumulate in the same immune environments as their Th cell counterparts and selectively inhibit specific Th response modules.

### Plasticity of Th17 and other Th lymphocytes

Th17 lymphocytes that express IL-17 and IFN-γ and that are transcriptionally similar to murine pathogenic Th17 lymphocytes were found at sites of inflammation in humans (158). In mice, it was shown that although both types of Th17 cells produced IL-17, non-pathogenic Th17 lymphocytes additionally expressed the immunoregulatory genes Il10, Il9, Maf, and Ahr, whereas expression of pro-inflammatory genes including *Csf2, Ifng, Tbx21, IL23r, and Gzmb* was upregulated in pathogenic Th17 (190, 191). IL-6 induces early expression of IL-1R required for early differentiation of Th17 cells *in vivo* (192). In addition, they differ in generation conditions: highly pathogenic Th17 lymphocytes are generated using a combination of IL-6 and IL-23 with IL-1β or TGFβ (190, 193).

Pathogenic Th17 lymphocytes exhibit dual Th1/Th17 positive characteristics, co-express RORγt and T-bet, as well as IL-12Rβ2 and IL-23R (194, 195). They are characterized by co-expression of pro-inflammatory cytokines such as granulocyte-macrophage colony-stimulating factor (GM-CSF), IFN-γ, IL-26, CCL20, and IL-22 (196–198). Phenotypic instability is thought to predispose Th17 cells to acquire a pro-inflammatory phenotype in chronically inflamed tissues.

Mycobacterium tuberculosis induces a Th1 population that co-expresses T-bet and RORγt, CXCR3, and CCR6 and produces IFN-γ (but not IL-17) (199). In addition, patients with RORC mutations lack Th1 (199), suggesting that they may originate from Th17 cells via plastic events in an IL-12-, TNF-α-, and/or IL-1β-dependent manner (200). Reverse trans-differentiation was shown. Non-classical CD161+CCR6+ Th1 lymphocytes of rheumatoid arthritis patients, unlike classical CD161-CCR6- Th1 cells, are reprogrammed into pathogenic Th17 lymphocytes in Th17-inducing conditions (127), which may contribute to their pathogenicity during the course of rheumatoid arthritis.

Circulating CD4+ memory T cells producing both IL-4 and IL-17, as well as IL-23R and CCR6, GATA3, and RORγt, were identified in patients with allergic asthma (201, 202) and with palmoplantar pustulosis (203). However, it is still unclear whether Th17/Th2 cells originate from Th17 or Th2 and whether IL-4 (Th17 to Th2 cell translation) or IL-1β, IL-6, IL-21 (Th2 to Th17 cell translation) occurs or whether additional cytokines are required.

A fraction of circulating human memory Tfh cells express CXCR5 and CCR6, Bcl6, and RORγt and produce IL-21, IL-22, and IL-17 (hence termed Tfh17) (204, 205). It remains to be clarified whether Tfh17 cells originate from Tfh or Th17 cells. Th17-to-Tfh plasticity may be relevant for Th17-mediated autoantibody generation.

Many different T cells with specific cytokine profiles are required for defense against various pathogenic exo- and endogenous influences. CD4+ T-lymphocyte subpopulations have varied degrees of plasticity and the ability to acquire new characteristics during the immune response. Even recognized terminally differentiated Th1/Th2 lymphocytes under certain conditions can transdifferentiate not only into each other but also into other subpopulations of Th lymphocytes. Th17 lymphocytes are characterized by a significant degree of instability; this is a highly heterogeneous subpopulation of effector cells whose protective role in inflammatory diseases remains to be studied. Treg cells, despite their isolated role in suppressing inflammatory reactions, also show features of plasticity and the possibility of transition to other subpopulations. In general, Treg cell plasticity is justified by the need to control different types of immune responses. Another important but poorly understood aspect of T cell plasticity is how different tissue microenvironments affect the differentiation and stability of human T cells. Determining the relative plasticity or stability of T cell subsets in different tissues is important for future therapeutic interventions in immune pathologies as diverse as chronic viral infections, cancer, and autoimmune diseases.

## Conclusion

Decades of research on T-helper cells, using modern research tools such as ChiPseq, RNAseq, and scRNAseq, demonstrated the diversity and heterogeneity of cell subpopulations, which provides the ability to respond rapidly and successfully to various challenges. T-helper cells are characterized by susceptibility to many exogenous and endogenous signals that can alter existing transcriptional programs, resulting in changes in the identity of T-helper cell subpopulations. It became evident that basic transcriptional networks could be cross-regulated and cross-expressed, creating unique subpopulations of T lymphocytes required for specific patterns of stimulation. Maintenance of tissue homeostasis and intracellular metabolism is closely linked to the stabilization of T cell subpopulations. Unraveling these complex mechanisms of plasticity and flexibility of T-helper cells and the conditions of their maintenance will allow for the generation of specific subpopulations of antigen-specific T-helper cells, regulating and controlling the type of immune response under a specific antigen. In the future, this could be a powerful tool for discovering new targets and optimizing existing therapies for specific patients and/or specific diseases.

## Author contributions

JK: Investigation, Writing – original draft. SS: Conceptualization, Writing – review & editing.

## Glossary

**Table:**

AP-1	activating protein-1
BATF	Basic leucine zipper transcription factor
Bcl2/Bcl6	B-cell CLL/lymphoma 2/6
Blimp1	B-lymphocyte-induced maturation protein 1
CCR	CC chemokines receptor
CXCR	CXC chemokines receptor
DGK	Diacylglycerol kinase
DNA	Deoxyribonucleic acid
DUSP2	Dual Specificity Phosphatase 2
EOMES	eomesodermin
ERK	extracellular signal-regulated kinase
FOXO	forkhead box O
FOXP3	forkhead box P3
GATA-3	GATA Binding Protein 3
GFI1	growth-factor independent 1
HIF1α	hypoxia-inducible factor 1α
IFN	interferon
*IKK*	IκB kinase
IL	interleukin
IP _3_	inositol 1, 4, 5-trisphosphate
IRF4	interferon-regulatory factor 4
ITAM	immunoreceptor tyrosine-based activation motif
Itk	interleukin-2-inducible T-cell kinase
iTreg	inducible regulatory T-cell
LAT	linker for activation of T cells
LMP7	low molecular weight polypeptide 7
MAF	macrophage-activating factor
MHC	major histocompatibility complex
mTOR	mammalian target of rapamycin
NFAT	Nuclear factor of activated T-cells
NF-κB	nuclear factor kappa-light-chain-enhancer of activated B cells
NR4A	nuclear receptor 4A
PKCθ	Protein kinase C theta
PP2A	Protein phosphatase 2A
RAR	retinoic acid receptor;
Ras	small GTFase, from **Ra**t **s**arcoma virus
ROR	retinoic acid receptor-related orphan receptor;
RUNX3	runt-related transcription factor 3
SHP	**S**rc-homology 2 domain-containing **p**rotein tyrosine **p**hosphatases
STAT	Signal transducer and activator of transcription
Stub1	STIP1 Homology And U-Box Containing Protein 1
T-bet	T-Cell-Specific T-Box Transcription Factor (or TBX21)
TCR	T cell receptor
Tfh	follicular helper T cell
TGF-β	transforming growth factor
Th	T-helper cell
TNF	tumor necrosis factor
Tregs	regulatory T-cells
TSC	tuberous sclerosis complex
Zap70	Zeta-chain-associated protein kinase 70

## References

[1] Rubtsov YP Niec RE Josefowicz S Li L Darce J Mathis D. Stability of the regulatory T cell lineage in vivo. Science (2010) 329(5999):1667–71. doi: 10.1126/science.1191996 PMC4262151 20929851

[2] Miyao T Floess S Setoguchi R Luche H Fehling HJ Waldmann H. Plasticity of foxp3+ T cells reflects promiscuous foxp3 expression in conventional T cells but not reprogramming of regulatory T cells. Immunity (2012) 36(2):262–75. doi: 10.1016/j.immuni.2011.12.012 22326580

[3] Kachler K Holzinger C Trufa DI Sirbu H Finotto S. The role of Foxp3 and Tbet co-expressing Treg cells in lung carcinoma. OncoImmunology (2018):e1456612. doi: 10.1080/2162402X.2018.1456612 30221050 PMC6136856

[4] Jiang C Wang H Xue M Lin L Wang J Cai G. Reprograming of peripheral Foxp3+ regulatory T cell towards Th17-like cell in patients with active systemic lupus erythematosus. Clin Immunol (2019) 209:108267. doi: 10.1016/j.clim.2019.108267 31639448

[5] Bhaumik S Mickael ME Moran M Spell M Basu R. RORγt promotes foxp3 expression by antagonizing the effector program in colonic regulatory T cells. J Immunol (2021) 207(8):2027–38. doi: 10.4049/jimmunol.2100175 PMC8490938 34518282

[6] Martínez-Blanco M Lozano-Ojalvo D Pérez-Rodríguez L Benedé S Molina E López-Fandiño R. Retinoic acid induces functionally suppressive foxp3+RORγt+ T cells *in vitro* . Front Immunol (2021) 12:675733. doi: 10.3389/fimmu.2021.675733 34447371 PMC8382797

[7] Lin H Wang H Liu Q Wang Z Wen S Wang L. A novel strategy to investigate the factors regulating the Treg to Tfr transition during acute viral infection. J Immunol Methods (2022) 505:113266. doi: 10.1016/j.jim.2022.113266 35398062

[8] Chen Y Zander R Khatun A Schauder DM Cui W. Transcriptional and epigenetic regulation of effector and memory CD8 T cell differentiation. Front Immunol (2018) 9:2826/BIBTEX. doi: 10.3389/FIMMU.2018.02826/BIBTEX 30581433 PMC6292868

[9] Kurd NS He Z Louis TL Milner JJ Omilusik KD Jin W. Early precursors and molecular determinants of tissue-resident memory CD8+ T lymphocytes revealed by single-cell RNA sequencing. Sci Immunol (2020) 5(47):1–36. doi: 10.1126/sciimmunol.aaz6894 PMC7341730 32414833

[10] Solouki S Huang W Elmore J Limper C Huang F August A. TCR signal strength and antigen affinity regulate CD8+ Memory T cells. J Immunol (2020) 205(5):1217–27. doi: 10.4049/jimmunol.1901167 PMC8104072 32759295

[11] Crowl JT Heeg M Ferry A Milner JJ Omilusik KD Toma C. Tissue-resident memory CD8+ T cells possess unique transcriptional, epigenetic and functional adaptations to different tissue environments. Nat Immunol (2022) 23(7):1121–31. doi: 10.1038/s41590-022-01229-8 PMC10041538 35761084

[12] Gagliani N Vesely MCA Iseppon A Brockmann L Xu H Palm NW. Th17 cells transdifferentiate into regulatory T cells during resolution of inflammation. Nature (2015) 523(7559):221–5. doi: 10.1038/nature14452 PMC4498984 25924064

[13] Akhmetzyanova I Zelinskyy G Littwitz-Salomon E Malyshkina A Dietze KK Streeck H. CD137 agonist therapy can reprogram regulatory T cells into cytotoxic CD4+ T cells with antitumor activity. J Immunol (2016) 196(1):484–92. doi: 10.4049/jimmunol.1403039 26608920

[14] Coit P Dozmorov MG Merrill JT McCune WJ Maksimowicz-McKinnon K Wren JD. Epigenetic reprogramming in naive CD4+ T cells favoring T cell activation and non-th1 effector T cell immune response as an early event in lupus flares. Arthritis Rheumatol (2016) 68(9):2200–9. doi: 10.1002/art.39720 PMC5001909 27111767

[15] Proserpio V Piccolo A Haim-Vilmovsky L Kar G Lönnberg T Svensson V. Single-cell analysis of CD4+ T-cell differentiation reveals three major cell states and progressive acceleration of proliferation. Genome Biol (2016) 17(1):103. doi: 10.1186/s13059-016-0957-5 27176874 PMC4866375

[16] Liu Z-Z Sun G-Q Hu X-H Kwak-Kim J Liao A-H. The transdifferentiation of regulatory T and Th17 cells in autoimmune/inflammatory diseases and its potential implications in pregnancy complications. Am J Reprod Immunol (2017) 78(2):e12657. doi: 10.1111/aji.12657 28251714

[17] Alterauge D Bagnoli JW Dahlström F Bradford BM Mabbott NA Buch T. Continued Bcl6 Expression Prevents the Transdifferentiation of Established Tfh Cells into Th1 Cells during Acute Viral Infection. Cell Rep (2020) 33(1):108232. doi: 10.1016/j.celrep.2020.108232 33027650

[18] Di Y Zhang M Chen Y Sun R Shen M Tian F. Catalpol inhibits tregs-to-th17 cell transdifferentiation by up-regulating let-7g-5p to reduce STAT3 protein levels. Yonsei Med J (2022) 63(1):56. doi: 10.3349/ymj.2022.63.1.56 34913284 PMC8688372

[19] Zhu J Paul WE. Peripheral CD4+ T-cell differentiation regulated by networks of cytokines and transcription factors. Immunol Rev (2010) 238(1):247–62. doi: 10.1111/j.1600-065X.2010.00951.x PMC2975272 20969597

[20] Gaud G Lesourne R Love PE. Regulatory mechanisms in T cell receptor signalling. Nat Rev Immunol (2018) 18(8):485–97. doi: 10.1038/s41577-018-0020-8 29789755

[21] Iborra S Ramos M Arana DM Lázaro S Aguilar F Santos E. N-ras couples antigen receptor signaling to eomesodermin and to functional CD8+ t cell memory but not to effector differentiation. J Exp Med (2013) 210(7):1463–79. doi: 10.1084/jem.20112495 PMC3698526 23776078

[22] Bertin S Lozano-Ruiz B Bachiller V García-Martínez I Herdman S Zapater P. Dual-specificity phosphatase 6 regulates CD4+ t-cell functions and restrains spontaneous colitis in IL-10-deficient mice. Mucosal Immunol (2015) 8(3):505–15. doi: 10.1038/mi.2014.84 PMC4363301 25227984

[23] Park S-H Rhee J Kim S-K Kang J-A Kwak J-S Son Y-O. BATF regulates collagen-induced arthritis by regulating t helper cell differentiation. Arthritis Res Ther (2018) 20(1):161. doi: 10.1186/s13075-018-1658-0 30071881 PMC6090970

[24] Carr TM Wheaton JD Houtz GM Ciofani M. JunB promotes Th17 cell identity and restrains alternative CD4+ t-cell programs during inflammation. Nat Commun (2017) 8(1):301. doi: 10.1038/s41467-017-00380-3 28824171 PMC5563507

[25] Hogan PG. Calcium–NFAT transcriptional signalling in t cell activation and t cell exhaustion. Cell Calcium (2017) 63:66–9. doi: 10.1016/j.ceca.2017.01.014 PMC5739523 28153342

[26] Joseph N Reicher B Barda-Saad M. The calcium feedback loop and t cell activation: How cytoskeleton networks control intracellular calcium flux. Biochim Biophys Acta (BBA) - Biomembranes (2014) 1838(2):557–68. doi: 10.1016/j.bbamem.2013.07.009 23860253

[27] Oh-hora M Rao A. Calcium signaling in lymphocytes. Curr Opin Immunol (2008) 20(3):250–8. doi: 10.1016/j.coi.2008.04.004 PMC2574011 18515054

[28] Oh-hora M. The calcium/NFAT pathway: role in development and function of regulatory t cells. Microbes Infection (2009) 11(5):612–9. doi: 10.1016/j.micinf.2009.04.008 PMC2696553 19375514

[29] Arendt CW Albrecht B Soos TJ Littman DR. Protein kinase c-θ: signaling from the center of the t-cell synapse. Curr Opin Immunol (2002) 14(3):323–30. doi: 10.1016/S0952-7915(02)00346-1 11973130

[30] Liu Y Witte S Liu Y-C Doyle M Elly C Altman A. Regulation of protein kinase cθ function during t cell activation by lck-mediated tyrosine phosphorylation. J Biol Chem (2000) 275(5):3603–9. doi: 10.1074/jbc.275.5.3603 10652356

[31] Monks CRF Kupfer H Tamir I Barlow A Kupfer A. Selective modulation of protein kinase c-Θ during t-cell activation. Nature (1997) 385(6611):83–6. doi: 10.1038/385083a0 8985252

[32] Pfeifhofer C Kofler K Gruber T Ghaffari Tabrizi N Lutz C Maly K. Protein kinase c θ affects Ca2+ mobilization and NFAT activation in primary mouse t cells. J Exp Med (2003) 197(11):1525–35. doi: 10.1084/jem.20020234 PMC2193906 12782715

[33] Sun Z Arendt CW Ellmeier W Schaeffer EM Sunshine MJ Gandhi L. PKC-θ is required for TCR-induced NF-κB activation in mature but not immature t lymphocytes. Nature (2000) 404(6776):402–7. doi: 10.1038/35006090 10746729

[34] Thome M. CARMA1, BCL-10 and MALT1 in lymphocyte development and activation. Nat Rev Immunol (2004) 4(5):348–59. doi: 10.1038/nri1352 15122200

[35] Quann EJ Liu X Altan-Bonnet G Huse M. A cascade of protein kinase c isozymes promotes cytoskeletal polarization in t cells. Nat Immunol (2011) 12(7):647–54. doi: 10.1038/ni.2033 PMC3119370 21602810

[36] Kang J-A Jeong SP Park D Hayden MS Ghosh S Park S-G. Transition from heterotypic to homotypic PDK1 homodimerization is essential for TCR-mediated NF-κB activation. J Immunol (2013) 190(9):4508–15. doi: 10.4049/jimmunol.1202923 PMC3664235 23530144

[37] Kang J-A Choi H Yang T Cho SK Park Z-Y Park S-G. PKCθ-mediated PDK1 phosphorylation enhances t cell activation by increasing PDK1 stability. Molecules Cells (2017) 40(1):37–44. doi: 10.14348/molcells.2017.2236 28152304 PMC5303887

[38] Lin X O’Mahony A Mu Y Geleziunas R Greene WC. Protein kinase c-θ participates in NF-κB activation induced by CD3-CD28 costimulation through selective activation of IκB kinase β. Mol Cell Biol (2000) 20(8):2933–40. doi: 10.1128/MCB.20.8.2933-2940.2000 PMC85537 10733597

[39] Park S-G Schulze-Luehrman J Hayden MS Hashimoto N Ogawa W Kasuga M. The kinase PDK1 integrates t cell antigen receptor and CD28 coreceptor signaling to induce NF-κB and activate t cells. Nat Immunol (2009) 10(2):158–66. doi: 10.1038/ni.1687 PMC2768497 19122654

[40] Battaglia M Stabilini A Roncarolo M-G. Rapamycin selectively expands CD4+CD25+FoxP3+ regulatory t cells. Blood (2005) 105(12):4743–8. doi: 10.1182/blood-2004-10-3932 15746082

[41] Chen H Zhang L Zhang H Xiao Y Shao L Li H. Disruption of TSC1/2 signaling complex reveals a checkpoint governing thymic CD4 + CD25 + Foxp3 + regulatory t-cell development in mice. FASEB J (2013) 27(10):3979–90. doi: 10.1096/fj.13-235408 23882125

[42] Pollizzi KN Powell JD. Regulation of t cells by mTOR: the known knowns and the known unknowns. Trends Immunol (2015) 36(1):13–20. doi: 10.1016/j.it.2014.11.005 25522665 PMC4290883

[43] Mikhailik A Ford B Keller J Chen Y Nassar N Carpino N. A phosphatase activity of sts-1 contributes to the suppression of TCR signaling. Mol Cell (2007) 27(3):486–97. doi: 10.1016/j.molcel.2007.06.015 PMC2709417 17679096

[44] Nagaishi T Pao L Lin S-H Iijima H Kaser A Qiao S-W. SHP1 phosphatase-dependent t cell inhibition by CEACAM1 adhesion molecule isoforms. Immunity (2006) 25(5):769–81. doi: 10.1016/j.immuni.2006.08.026 17081782

[45] Paster W Bruger AM Katsch K Grégoire C Roncagalli R Fu G. A THEMIS : SHP 1 complex promotes t-cell survival. EMBO J (2015) 34(3):393–409. doi: 10.15252/embj.201387725 25535246 PMC4339124

[46] Lorenz U. SHP-1 and SHP-2 in t cells: two phosphatases functioning at many levels. Immunol Rev (2009) 228(1):342–59. doi: 10.1111/j.1600-065X.2008.00760.x PMC2669678 19290938

[47] Salmond RJ Huyer G Kotsoni A Clements L Alexander DR. The src homology 2 domain-containing tyrosine phosphatase 2 regulates primary t-dependent immune responses and th cell differentiation. J Immunol (2005) 175(10):6498–508. doi: 10.4049/jimmunol.175.10.6498 16272304

[48] Li Q-J Chau J Ebert PJR Sylvester G Min H Liu G. miR-181a is an intrinsic modulator of t cell sensitivity and selection. Cell (2007) 129(1):147–61. doi: 10.1016/j.cell.2007.03.008 17382377

[49] Hasegawa K Martin F Huang G Tumas D Diehl L Chan AC. PEST domain-enriched tyrosine phosphatase (PEP) regulation of Effector/Memory t cells. Science (2004) 303(5658):685–9. doi: 10.1126/science.1092138 14752163

[50] Chuang E Fisher TS Morgan RW Robbins MD Duerr JM Vander Heiden MG. The CD28 and CTLA-4 receptors associate with the Serine/Threonine phosphatase PP2A. Immunity (2000) 13(3):313–22. doi: 10.1016/S1074-7613(00)00031-5 11021529

[51] Lu D Liu L Ji X Gao Y Chen X Liu Y. The phosphatase DUSP2 controls the activity of the transcription activator STAT3 and regulates TH17 differentiation. Nat Immunol (2015) 16(12):1263–73. doi: 10.1038/ni.3278 26479789

[52] Twohig JP Cardus Figueras A Andrews R Wiede F Cossins BC Derrac Soria A. Activation of naïve CD4+ t cells re-tunes STAT1 signaling to deliver unique cytokine responses in memory CD4+ t cells. Nat Immunol (2019) 20(4):458–70. doi: 10.1038/s41590-019-0350-0 PMC7610646 30890796

[53] Vogel KU Edelmann SL Jeltsch KM Bertossi A Heger K Heinz GA. Roquin paralogs 1 and 2 redundantly repress the icos and Ox40 costimulator mRNAs and control follicular helper t cell differentiation. Immunity (2013) 38(4):655–68. doi: 10.1016/j.immuni.2012.12.004 23583643

[54] Kalim KW Basler M Kirk CJ Groettrup M. Immunoproteasome subunit LMP7 deficiency and inhibition suppresses Th1 and Th17 but enhances regulatory t cell differentiation. J Immunol (2012) 189(8):4182–93. doi: 10.4049/jimmunol.1201183 22984077

[55] Chen Z Barbi J Bu S Yang H-Y Li Z Gao Y. The ubiquitin ligase Stub1 negatively modulates regulatory t cell suppressive activity by promoting degradation of the transcription factor Foxp3. Immunity (2013) 39(2):272–85. doi: 10.1016/j.immuni.2013.08.006 PMC3817295 23973223

[56] Mueller DL. E3 ubiquitin ligases as t cell anergy factors. Nat Immunol (2004) 5(9):883–90. doi: 10.1038/ni1106 15334084

[57] Olenchock BA Guo R Carpenter JH Jordan M Topham MK Koretzky GA. Disruption of diacylglycerol metabolism impairs the induction of t cell anergy. Nat Immunol (2006) 7(11):1174–81. doi: 10.1038/ni1400 17028587

[58] Zhong X-P Guo R Zhou H Liu C Wan C-K. Diacylglycerol kinases in immune cell function and self-tolerance. Immunol Rev (2008) 224(1):249–64. doi: 10.1111/j.1600-065X.2008.00647.x PMC3342643 18759932

[59] Joshi RP Schmidt AM Das J Pytel D Riese MJ Lester M. The ζ isoform of diacylglycerol kinase plays a predominant role in regulatory t cell development and TCR-mediated ras signaling. Sci Signaling (2013) 6(303). doi: 10.1126/scisignal.2004373 PMC4096120 24280043

[60] Georgopoulos K Bigby M Wang J-H Molnar A Wu P Winandy S. The ikaros gene is required for the development of all lymphoid lineages. Cell (1994) 79(1):143–56. doi: 10.1016/0092-8674(94)90407-3 7923373

[61] Wang J-H Nichogiannopoulou A Wu L Sun L Sharpe AH Bigby M. Selective defects in the development of the fetal and adult lymphoid system in mice with an ikaros null mutation. Immunity (1996) 5(6):537–49. doi: 10.1016/S1074-7613(00)80269-1 8986714

[62] Ting C-N Olson MC Barton KP Leiden JM. Transcription factor GATA-3 is required for development of the t-cell lineage. Nature (1996) 384(6608):474–8. doi: 10.1038/384474a0 8945476

[63] Zhang D-H Cohn L Ray P Bottomly K Ray A. Transcription factor GATA-3 is differentially expressed in murine Th1 and Th2 cells and controls Th2-specific expression of the interleukin-5 gene. J Biol Chem (1997) 272(34):21597–603. doi: 10.1074/jbc.272.34.21597 9261181

[64] Zheng W Flavell RA. The transcription factor GATA-3 is necessary and sufficient for Th2 cytokine gene expression in CD4 t cells. Cell (1997) 89(4):587–96. doi: 10.1016/S0092-8674(00)80240-8 9160750

[65] Taniuchi I. CD4 helper and CD8 cytotoxic t cell differentiation. Annu Rev Immunol (2018) 36(1):579–601. doi: 10.1146/annurev-immunol-042617-053411 29677476

[66] Koch U Lacombe TA Holland D Bowman JL Cohen BL Egan SE. Subversion of the T/B lineage decision in the thymus by lunatic fringe-mediated inhibition of notch-1. Immunity (2001) 15(2):225–36. doi: 10.1016/S1074-7613(01)00189-3 11520458

[67] Laky K Fleischacker C Fowlkes BJ. TCR and notch signaling in CD4 and CD8 t-cell development. Immunol Rev (2006) 209(1):274–83. doi: 10.1111/j.0105-2896.2006.00358.x 16448548

[68] Choi YS Eto D Yang JA Lao C Crotty S. Cutting edge: STAT1 is required for IL-6–mediated Bcl6 induction for early follicular helper cell differentiation. J Immunol (2013) 190(7):3049–53. doi: 10.4049/jimmunol.1203032 PMC3626564 23447690

[69] Harrington LE Hatton RD Mangan PR Turner H Murphy TL Murphy KM. Interleukin 17–producing CD4+ effector t cells develop *via* a lineage distinct from the t helper type 1 and 2 lineages. Nat Immunol (2005) 6(11):1123–32. doi: 10.1038/ni1254 16200070

[70] Afkarian M Sedy JR Yang J Jacobson NG Cereb N Yang SY. T-bet is a STAT1-induced regulator of IL-12R expression in naïve CD4+ t cells. Nat Immunol (2002) 3(6):549–57. doi: 10.1038/ni794 12006974

[71] Szabo SJ Kim ST Costa GL Zhang X Fathman CG Glimcher LH. A novel transcription factor, t-bet, directs Th1 lineage commitment. Cell (2000) 100(6):655–69. doi: 10.1016/S0092-8674(00)80702-3 10761931

[72] Chang H Zhao F Xie X Liao Y Song Y Liu C. PPARα suppresses Th17 cell differentiation through IL-6/STAT3/RORγt pathway in experimental autoimmune myocarditis. Exp Cell Res (2019) 375(1):22–30. doi: 10.1016/j.yexcr.2018.12.005 30557558

[73] Kaplan MH Sun Y-L Hoey T Grusby MJ. Impaired IL-12 responses and enhanced development of Th2 cells in Stat4-deficient mice. Nature (1996) 382(6587):174–7. doi: 10.1038/382174a0 8700209

[74] Thierfelder WE van Deursen JM Yamamoto K Tripp RA Sarawar SR Carson RT. Requirement for Stat4 in interleukin-12-mediated responses of natural killer and t cells. Nature (1996) 382(6587):171–4. doi: 10.1038/382171a0 8700208

[75] Buzzelli AA McWilliams IL Shin B Bryars MT Harrington LE. Intrinsic STAT4 expression controls effector CD4 t cell migration and Th17 pathogenicity. J Immunol (2023) 210(11):1667–76. doi: 10.4049/jimmunol.2200606 PMC11302403 37093664

[76] Bassil R Orent W Olah M Kurdi AT Frangieh M Buttrick T. BCL6 controls Th9 cell development by repressing Il9 transcription. J Immunol (2014) 193(1):198–207. doi: 10.4049/jimmunol.1303184 24879792 PMC4130220

[77] Cote-Sierra J Foucras G Guo L Chiodetti L Young HA Hu-Li J. Interleukin 2 plays a central role in Th2 differentiation. Proc Natl Acad Sci (2004) 101(11):3880–5. doi: 10.1073/pnas.0400339101 PMC374338 15004274

[78] Ji L-S Sun X-H Zhang X Zhou Z-H Yu Z Zhu X-J. Mechanism of follicular helper t cell differentiation regulated by transcription factors. J Immunol Res (2020) 2020:1–9. doi: 10.1155/2020/1826587 PMC7387970 32766317

[79] Johnston RJ Choi YS Diamond JA Yang JA Crotty S. STAT5 is a potent negative regulator of TFH cell differentiation. J Exp Med (2012) 209(2):243–50. doi: 10.1084/jem.20111174 PMC3281266 22271576

[80] Kagami S Nakajima H Suto A Hirose K Suzuki K Morita S. Stat5a regulates t helper cell differentiation by several distinct mechanisms. Blood (2001) 97(8):2358–65. doi: 10.1182/blood.V97.8.2358 11290598

[81] Liao W Spolski R Li P Du N West EE Ren M. Opposing actions of IL-2 and IL-21 on Th9 differentiation correlate with their differential regulation of BCL6 expression. Proc Natl Acad Sci (2014) 111(9):3508–13. doi: 10.1073/pnas.1301138111 PMC3948278 24550509

[82] Nurieva RI Podd A Chen Y Alekseev AM Yu M Qi X. STAT5 protein negatively regulates t follicular helper (Tfh) cell generation and function. J Biol Chem (2012) 287(14):11234–9. doi: 10.1074/jbc.M111.324046 PMC3322890 22318729

[83] Yao Z Kanno Y Kerenyi M Stephens G Durant L Watford WT. Nonredundant roles for Stat5a/b in directly regulating Foxp3. Blood (2007) 109(10):4368–75. doi: 10.1182/blood-2006-11-055756 PMC1885496 17227828

[84] Kaplan MH Schindler U Smiley ST Grusby MJ. Stat6 is required for mediating responses to IL-4 and for the development of Th2 cells. Immunity (1996) 4(3):313–9. doi: 10.1016/S1074-7613(00)80439-2 8624821

[85] Maier E Duschl A Horejs-Hoeck J. STAT6-dependent and -independent mechanisms in Th2 polarization. Eur J Immunol (2012) 42(11):2827–33. doi: 10.1002/eji.201242433 PMC3557721 23041833

[86] Takeda K Tanaka T Shi W Matsumoto M Minami M Kashiwamura S. Essential role of Stat6 in IL-4 signalling. Nature (1996) 380(6575):627–30. doi: 10.1038/380627a0 8602263

[87] Arroyo-Olarte RD Rivera-Rugeles A Nava-Lira E Sánchez-Barrera Á Ledesma-Soto Y Saavedra R. STAT6 controls the stability and suppressive function of regulatory t cells. Eur J Immunol (2023) 53(5). doi: 10.1002/eji.202250128 36785881

[88] Delgado-Ramirez Y Ocaña-Soriano A Ledesma-Soto Y Olguín JE Hernandez-Ruiz J Terrazas LI. STAT6 is critical for the induction of regulatory t cells *In vivo* controlling the initial steps of colitis-associated cancer. Int J Mol Sci (2021) 22(8):4049. doi: 10.3390/ijms22084049 33919941 PMC8070924

[89] Crotty S Johnston RJ Schoenberger SP. Effectors and memories: Bcl-6 and blimp-1 in t and b lymphocyte differentiation. Nat Immunol (2010) 11(2):114–20. doi: 10.1038/ni.1837 PMC2864556 20084069

[90] Johnston RJ Poholek AC DiToro D Yusuf I Eto D Barnett B. Bcl6 and blimp-1 are reciprocal and antagonistic regulators of t follicular helper cell differentiation. Science (2009) 325(5943):1006–10. doi: 10.1126/science.1175870 PMC2766560 19608860

[91] Nurieva RI Chung Y Martinez GJ Yang XO Tanaka S Matskevitch TD. Bcl6 mediates the development of t follicular helper cells. Science (2009) 325(5943):1001–5. doi: 10.1126/science.1176676 PMC2857334 19628815

[92] Yu D Rao S Tsai LM Lee SK He Y Sutcliffe EL. The transcriptional repressor bcl-6 directs t follicular helper cell lineage commitment. Immunity (2009) 31(3):457–68. doi: 10.1016/j.immuni.2009.07.002 19631565

[93] Tang W Wang H Murphy PM Siebenlist U. Bcl-3 suppresses Th9 differentiation by regulating glutamine utilization. BioRxiv (2021):2021.07.06.451316. doi: 10.1101/2021.07.06.451316

[94] Tsuda M Hamade H Thomas LS Salumbides BC Potdar AA Wong MH. A role for BATF3 in TH9 differentiation and t-cell-driven mucosal pathologies. Mucosal Immunol (2019) 12(3):644–55. doi: 10.1038/s41385-018-0122-4 PMC6462229 30617301

[95] Durant L Watford WT Ramos HL Laurence A Vahedi G Wei L. Diverse targets of the transcription factor STAT3 contribute to t cell pathogenicity and homeostasis. Immunity (2010) 32(5):605–15. doi: 10.1016/j.immuni.2010.05.003 PMC3148263 20493732

[96] Chakraborty AK Weiss A. Insights into the initiation of TCR signaling. Nat Immunol (2014) 15(9):798–807. doi: 10.1038/ni.2940 25137454 PMC5226627

[97] Courtney AH Lo WL Weiss A. TCR signaling: mechanisms of initiation and propagation. Trends Biochem Sci (2018) 43(2):108–23. doi: 10.1016/j.tibs.2017.11.008 PMC5801066 29269020

[98] Bhattacharyya ND Feng CG. Regulation of T helper cell fate by TCR signal strength. Front Immunol (2020) 11:624/BIBTEX. doi: 10.3389/FIMMU.2020.00624/BIBTEX 32508803 PMC7248325

[99] Hwang J-R Byeon Y Kim D Park S-G. Recent insights of T cell receptor-mediated signaling pathways for T cell activation and development. Exp Mol Med (2020) 52(5):750–61. doi: 10.1038/s12276-020-0435-8 PMC7272404 32439954

[100] Rogers PR Croft M. Peptide dose, affinity, and time of differentiation can contribute to the Th1/Th2 cytokine balance. J Immunol (Baltimore Md.: 1950) (1999) 163(3):1205–13. doi: 10.4049/jimmunol.163.3.1205 10415015

[101] Keck S Schmaler M Ganter S Wyss L Oberle S Huseby ES. Antigen affinity and antigen dose exert distinct influences on CD4 T-cell differentiation. Proc Natl Acad Sci (2014) 111(41):14852–7. doi: 10.1073/pnas.1403271111 PMC4205596 25267612

[102] van Panhuys N Klauschen F Germain RN. T-cell-receptor-dependent signal intensity dominantly controls CD4+ T cell polarization *in vivo* . Immunity (2014) 41(1):63–74. doi: 10.1016/j.immuni.2014.06.003 24981853 PMC4114069

[103] Yamane H Zhu J Paul WE. Independent roles for IL-2 and GATA-3 in stimulating naive CD4+ T cells to generate a Th2-inducing cytokine environment. J Exp Med (2005) 202(6):793–804. doi: 10.1084/JEM.20051304 16172258 PMC2212937

[104] Bhaumik S Basu R. Cellular and molecular dynamics of th17 differentiation and its developmental plasticity in the intestinal immune response. Front Immunol (2017) 8:254. doi: 10.3389/fimmu.2017.00254 28408906 PMC5374155

[105] Wyss L Stadinski BD King CG Schallenberg S McCarthy NI Lee JY. Affinity for self antigen selects Treg cells with distinct functional properties. Nat Immunol (2016) 17(9):1093–101. doi: 10.1038/ni.3522 PMC4994872 27478940

[106] This S Valbon SF Lebel M-È Melichar HJ. Strength and numbers: the role of affinity and avidity in the ‘Quality’ of T cell tolerance. Cells (2021) 10(6):1530. doi: 10.3390/cells10061530 34204485 PMC8234061

[107] Hogquist KA Jameson SC. The self-obsession of T cells: how TCR signaling thresholds affect fate “decisions” and effector function. Nat Immunol (2014) 15(9):815–23. doi: 10.1038/ni.2938 PMC4348363 25137456

[108] Klein L Kyewski B Allen PM Hogquist KA. Positive and negative selection of the T cell repertoire: what thymocytes see (and don’t see). Nat Rev Immunol (2014) 14(6):377–91. doi: 10.1038/nri3667 PMC4757912 24830344

[109] Kotov DI Mitchell JS Pengo T Ruedl C Way SS Langlois RA. TCR affinity biases th cell differentiation by regulating CD25, eef1e1, and gbp2. J Immunol (2019) 202(9):2535–45. doi: 10.4049/jimmunol.1801609 PMC6478541 30858199

[110] Micossé C von Meyenn L Steck O Kipfer E Adam C Simillion C. Human “TH9” cells are a subpopulation of PPAR-+ TH2 cells. Sci Immunol (2019) 4(31):5943. doi: 10.1126/SCIIMMUNOL.AAT5943/SUPPL_FILE/AAT5943_TABLE_S1.XLSX 30658968

[111] Schwartz DM Farley TK Richoz N Laurence A Meylan O O’ JJ. Retinoic acid receptor alpha represses a th9 transcriptional and epigenomic program to reduce allergic pathology. Immunity (2019) 50(1):106–20. doi: 10.1016/j.immuni.2018.12.014 PMC6338086 30650370

[112] Djuretic IM Levanon D Negreanu V Groner Y Rao A Ansel KM. Transcription factors T-bet and Runx3 cooperate to activate Ifng and silence Il4 in T helper type 1 cells. Nat Immunol (2007) 8(2):145–53. doi: 10.1038/ni1424 17195845

[113] Zhu J Yamane H Cote-Sierra J Guo L Paul WE. GATA-3 promotes Th2 responses through three different mechanisms: induction of Th2 cytokine production, selective growth of Th2 cells and inhibition of Th1 cell-specific factors. Cell Res (2006) 16(1):3–10. doi: 10.1038/sj.cr.7310002 16467870

[114] Goswami R Jabeen R Yagi R Pham D Zhu J Goenka S. STAT6-dependent regulation of th9 development. J Immunol (2012) 188(3):968–75. doi: 10.4049/jimmunol.1102840 PMC3262957 22180613

[115] Ivanov II McKenzie BS Zhou L Tadokoro CE Lepelley A Lafaille JJ. The orphan nuclear receptor RORγt directs the differentiation program of proinflammatory IL-17+ T helper cells. Cell (2006) 126(6):1121–33. doi: 10.1016/j.cell.2006.07.035 16990136

[116] Dong C. TH17 cells in development: an updated view of their molecular identity and genetic programming. Nat Rev Immunol (2008) 8(5):337–48. doi: 10.1038/nri2295 18408735

[117] Bedoya SK Wilson TD Collins EL Lau K Larkin J. Isolation and th17 differentiation of na&iuml;ve CD4 T lymphocytes. J Visualized Experiments (2013) 79:1–12. doi: 10.3791/50765 PMC3935776 24121559

[118] Xu L Kitani A Strober W. Molecular mechanisms regulating TGF-β-induced Foxp3 expression. Mucosal Immunol (2010) 3(3):230–8. doi: 10.1038/mi.2010.7 PMC3673708 20404810

[119] Eto D Lao C DiToro D Barnett B Escobar TC Kageyama R. IL-21 and IL-6 are critical for different aspects of B cell immunity and redundantly induce optimal follicular helper CD4 T cell (Tfh) differentiation. PloS One (2011) 6(3):e17739. doi: 10.1371/journal.pone.0017739 21423809 PMC3056724

[120] Jia L Wu C. The biology and functions of th22 cells. In: Sun B (eds) T Helper Cell Differentiation and Their Function. Advances in Experimental Medicine and Biology. Dordrecht: Springer. (2014) 841:209–30. doi: 10.1007/978-94-017-9487-9_8 25261209

[121] Radens CM Blake D Jewell P Barash Y Lynch KW. Meta-analysis of transcriptomic variation in T-cell populations reveals both variable and consistent signatures of gene expression and splicing. RNA (2020) 26(10):1320–33. doi: 10.1261/rna.075929.120 PMC7491319 32554554

[122] Martinez-Sanchez ME Mendoza L Villarreal C Alvarez-Buylla ER. A minimal regulatory network of extrinsic and intrinsic factors recovers observed patterns of CD4+ T cell differentiation and plasticity. PloS Comput Biol (2015) 11(6):e1004324. doi: 10.1371/journal.pcbi.1004324 26090929 PMC4475012

[123] Oja AE Piet B van der Zwan D Blaauwgeers H Mensink M de Kivit S. Functional heterogeneity of CD4+ Tumor-infiltrating lymphocytes with a resident memory phenotype in NSCLC. Front Immunol (2018) 9:2654. doi: 10.3389/fimmu.2018.02654 30505306 PMC6250821

[124] Geginat J Paroni M Maglie S Alfen JS Kastirr I Gruarin P. Plasticity of human CD4 T cell subsets. Front Immunol (2014) 5:630. doi: 10.3389/fimmu.2014.00630 25566245 PMC4267263

[125] Kleinewietfeld M Hafler DA. The plasticity of human Treg and Th17 cells and its role in autoimmunity. Semin Immunol (2013) 25(4):305–12. doi: 10.1016/j.smim.2013.10.009 PMC3905679 24211039

[126] Cerboni S Gehrmann U Preite S Mitra S. Cytokine-regulated Th17 plasticity in human health and diseases. Immunology (2021) 163(1):3–18. doi: 10.1111/imm.13280 33064842 PMC8044333

[127] Leipe J Pirronello F Klose A Schulze-Koops H Skapenko A. Increased plasticity of non-classic Th1 cells toward the Th17 phenotype. Modern Rheumatol (2020) 30(5):930–6. doi: 10.1080/14397595.2019.1667473 31512538

[128] Bartsch P Kilian C Hellmig M Paust H-J Borchers A Sivayoganathan A. Th17 cell plasticity towards a T-bet-dependent Th1 phenotype is required for bacterial control in Staphylococcus aureus infection. PloS Pathog (2022) 18(4):e1010430. doi: 10.1371/journal.ppat.1010430 35446923 PMC9064098

[129] Sundrud MS Grill SM Ni D Nagata K Alkan SS Subramaniam A. Genetic reprogramming of primary human T cells reveals functional plasticity in th cell differentiation. J Immunol (2003) 171(7):3542–9. doi: 10.4049/jimmunol.171.7.3542 14500650

[130] Krawczyk CM Shen H Pearce EJ. Functional plasticity in memory T helper cell responses. J Immunol (2007) 178(7):4080–8. doi: 10.4049/jimmunol.178.7.4080 17371962

[131] Duddu AS Majumdar SS Sahoo S Jhunjhunwala S Jolly MK. Emergent dynamics of a three-node regulatory network explain phenotypic switching and heterogeneity: a case study of Th1/Th2/Th17 cell differentiation. Mol Biol Cell (2022) 33(6):1–14. doi: 10.1091/mbc.E21-10-0521 PMC9265159 35353012

[132] Renaude E Kroemer M Borg C Peixoto P Hervouet E Loyon R. Epigenetic reprogramming of CD4+ Helper T cells as a strategy to improve anticancer immunotherapy. Front Immunol (2021) 12:669992. doi: 10.3389/fimmu.2021.669992 34262562 PMC8273698

[133] Chang S Collins PL Aune TM. T-bet dependent removal of sin3A-histone deacetylase complexes at the *ifng* locus drives th1 differentiation. J Immunol (2008) 181(12):8372–81. doi: 10.4049/jimmunol.181.12.8372 PMC2794428 19050254

[134] Tumes DJ Onodera A Suzuki A Shinoda K Endo Y Iwamura C. The polycomb protein ezh2 regulates differentiation and plasticity of CD4+ T helper type 1 and type 2 cells. Immunity (2013) 39(5):819–32. doi: 10.1016/j.immuni.2013.09.012 24238339

[135] Li Q Zou J Wang M Ding X Chepelev I Zhou X. Critical role of histone demethylase Jmjd3 in the regulation of CD4+ T-cell differentiation. Nat Commun (2014) 5(1):5780. doi: 10.1038/ncomms6780 25531312 PMC4274750

[136] Perez VL Lederer JA Lichtman AH Abbas AK. Stability of T _h_ 1 and T _h_ 2 populations. Int Immunol (1995) 7(5):869–75. doi: 10.1093/intimm/7.5.869 7547713

[137] Panzer M Sitte S Wirth S Drexler I Sparwasser T Voehringer D. Rapid *In Vivo* Conversion of Effector T Cells into Th2 Cells during Helminth Infection. J Immunol (2012) 188(2):615–23. doi: 10.4049/jimmunol.1101164 22156341

[138] Murphy E Shibuya K Hosken N Openshaw P Maino V Davis K. Reversibility of T helper 1 and 2 populations is lost after long-term stimulation. J Exp Med (1996) 183(3):901–13. doi: 10.1084/jem.183.3.901 PMC2192360 8642294

[139] Murphy KM Stockinger B. Effector T cell plasticity: flexibility in the face of changing circumstances. Nat Immunol (2010) 11(8):674–80. doi: 10.1038/ni.1899 PMC3249647 20644573

[140] Hegazy AN Peine M Helmstetter C Panse I Fröhlich A Bergthaler A. Interferons direct th2 cell reprogramming to generate a stable GATA-3+T-bet+ Cell subset with combined th2 and th1 cell functions. Immunity (2010) 32(1):116–28. doi: 10.1016/j.immuni.2009.12.004 20079668

[141] Ankathatti Munegowda M Xu S Freywald A Xiang J. CD4+ Th2 cells function alike effector Tr1 and Th1 cells through the deletion of a single cytokine IL-6 and IL-10 gene. Mol Immunol (2012) 51(2):143–9. doi: 10.1016/j.molimm.2012.02.120 22424785

[142] Nagaoka M Hatta Y Kawaoka Y Malherbe LP. Antigen Signal Strength during Priming Determines Effector CD4 T Cell Function and Antigen Sensitivity during Influenza Virus Challenge. J Immunol (2014) 193(6):2812–20. doi: 10.4049/jimmunol.1401358 PMC4157108 25086170

[143] Ahmadzadeh M Farber DL. Functional plasticity of an antigen-specific memory CD4 T cell population. Proc Natl Acad Sci (2002) 99(18):11802–7. doi: 10.1073/pnas.192263099 PMC129349 12192093

[144] Miller AT Wilcox HM Lai Z Berg LJ. Signaling through Itk Promotes T Helper 2 Differentiation *via* Negative Regulation of T-bet. Immunity (2004) 21(1):67–80. doi: 10.1016/j.immuni.2004.06.009 15345221

[145] Veldhoen M Uyttenhove C van Snick J Helmby H Westendorf A Buer J. Transforming growth factor-β “reprograms” the differentiation of T helper 2 cells and promotes an interleukin 9–producing subset. Nat Immunol (2008) 9(12):1341–6. doi: 10.1038/ni.1659 18931678

[146] Umezu-Goto M Kajiyama Y Kobayashi N Kaminuma O Suko M Mori A. IL-9 production by peripheral blood mononuclear cells of atopic asthmatics. Int Arch Allergy Immunol (2007) 143(Suppl. 1):76–9. doi: 10.1159/000101410 17541282

[147] Tan C Aziz MK Lovaas JD Vistica BP Shi G Wawrousek EF. Antigen-specific th9 cells exhibit uniqueness in their kinetics of cytokine production and short retention at the inflammatory site. J Immunol (2010) 185(11):6795–801. doi: 10.4049/jimmunol.1001676 PMC2988091 20971929

[148] Nakamura A Takahashi D Nakamura Y Yamada T Matsumoto M Hase K. Polyamines polarized Th2/Th9 cell-fate decision by regulating GATA3 expression. Arch Biochem Biophysics (2020) 693:108587. doi: 10.1016/j.abb.2020.108587 32946839

[149] Abdelaziz M Wang H Cheng J Xu H. Th2 cells as an intermediate for the differentiation of naive T cells into Th9 cells, associated with the Smad3/Smad4 and IRF4 pathway. Exp Ther Med (2020) 19:1947–54. doi: 10.3892/etm.2020.8420 PMC7027129 32104253

[150] Khokhar M Purohit P Gadwal A Tomo S Bajpai NK Shukla R. The differentially expressed genes responsible for the development of T helper 9 cells from T helper 2 cells in various disease states: immuno-interactomics study. JMIR Bioinf Biotechnol (2023) 4:e42421. doi: 10.2196/42421 PMC11135241 38935935

[151] Zaretsky AG Taylor JJ King IL Marshall FA Mohrs M Pearce EJ. T follicular helper cells differentiate from Th2 cells in response to helminth antigens. J Exp Med (2009) 206(5):991–9. doi: 10.1084/jem.20090303 PMC2715032 19380637

[152] Ballesteros-Tato A Randall TD Lund FE Spolski R Leonard WJ León B. T follicular helper cell plasticity shapes pathogenic T helper 2 cell-mediated immunity to inhaled house dust mite. Immunity (2016) 44(2):259–73. doi: 10.1016/j.immuni.2015.11.017 PMC4758890 26825674

[153] Lu KT Kanno Y Cannons JL Handon R Bible P Elkahloun AG. Functional and epigenetic studies reveal multistep differentiation and plasticity of *in vitro*-generated and *in vivo*-derived follicular T helper cells. Immunity (2011) 35(4):622–32. doi: 10.1016/j.immuni.2011.07.015 PMC3235706 22018472

[154] Solé P Yamanouchi J Garnica J Uddin MM Clarke R Moro J. A T follicular helper cell origin for T regulatory type 1 cells. Cell Mol Immunol (2023) 20(5):489–511. doi: 10.1038/s41423-023-00989-z 36973489 PMC10202951

[155] Saraiva M Christensen JR Veldhoen M Murphy TL Murphy KM O’Garra A. Interleukin-10 production by th1 cells requires interleukin-12-induced STAT4 transcription factor and ERK MAP kinase activation by high antigen dose. Immunity (2009) 31(2):209–19. doi: 10.1016/j.immuni.2009.05.012 PMC2791889 19646904

[156] Cope A Le Friec G Cardone J Kemper C. The Th1 life cycle: molecular control of IFN-γ to IL-10 switching. Trends Immunol (2011) 32(6):278–86. doi: 10.1016/j.it.2011.03.010 21531623

[157] Parish IA Marshall HD Staron MM Lang PA Brüstle A Chen JH. Chronic viral infection promotes sustained Th1-derived immunoregulatory IL-10 *via* BLIMP-1. J Clin Invest (2014) 124(8):3455–68. doi: 10.1172/JCI66108 PMC4109559 25003188

[158] Mills KHG. IL-17 and IL-17-producing cells in protection versus pathology. Nat Rev Immunol (2023) 23(1):38–54. doi: 10.1038/s41577-022-00746-9 35790881 PMC9255545

[159] Voo KS Wang Y-H Santori FR Boggiano C Wang Y-H Arima K. Identification of IL-17-producing FOXP3 ^+^ regulatory T cells in humans. Proc Natl Acad Sci (2009) 106(12):4793–8. doi: 10.1073/pnas.0900408106 PMC2653560 19273860

[160] Zhu L Song H Zhang L Meng H. Characterization of IL-17-producing Treg cells in type 2 diabetes patients. Immunologic Res (2019) 67(4–5):443–9. doi: 10.1007/s12026-019-09095-7 31713831

[161] Furuyama K Kondo Y Shimizu M Yokosawa M Segawa S Iizuka A. RORγt+Foxp3+ regulatory T cells in the regulation of autoimmune arthritis. Clin Exp Immunol (2022) 207(2):176–87. doi: 10.1093/cei/uxab007 PMC8982961 35020849

[162] Kim J Moreno A Krueger JG. The imbalance between Type 17 T-cells and regulatory immune cell subsets in psoriasis vulgaris. Front Immunol (2022) 13:1005115. doi: 10.3389/fimmu.2022.1005115 36110854 PMC9468415

[163] Lochner M Peduto L Cherrier M Sawa S Langa F Varona R. *In vivo* equilibrium of proinflammatory IL-17+ and regulatory IL-10+ Foxp3+ RORγt+ T cells. J Exp Med (2008) 205(6):1381–93. doi: 10.1084/jem.20080034 PMC2413035 18504307

[164] Yang XO Nurieva R Martinez GJ Kang HS Chung Y Pappu BP. Molecular antagonism and plasticity of regulatory and inflammatory T cell programs. Immunity (2008) 29(1):44–56. doi: 10.1016/j.immuni.2008.05.007 18585065 PMC2630532

[165] Tartar DM VanMorlan AM Wan X Guloglu FB Jain R Haymaker CL. FoxP3+RORγt+ T helper intermediates display suppressive function against autoimmune diabetes. J Immunol (2010) 184(7):3377–85. doi: 10.4049/jimmunol.0903324 PMC2843758 20181889

[166] Damasceno LEA Prado DS Veras FP Fonseca MM Toller-Kawahisa JE Rosa MH. PKM2 promotes Th17 cell differentiation and autoimmune inflammation by fine-tuning STAT3 activation. J Exp Med (2020) 217(10):2701–13. doi: 10.1084/jem.20190613 PMC7537396 32697823

[167] Minns D Smith KJ Alessandrini V Hardisty G Melrose L Jackson-Jones L. The neutrophil antimicrobial peptide cathelicidin promotes Th17 differentiation. Nat Commun (2021) 12(1):1285. doi: 10.1038/s41467-021-21533-5 33627652 PMC7904761

[168] Zhang X Zhang X Qiu C Shen H Zhang H He Z. The imbalance of Th17/Treg *via* STAT3 activation modulates cognitive impairment in *P. gingivalis* LPS-induced periodontitis mice. J Leukocyte Biol (2021) 110(3):511–24. doi: 10.1002/JLB.3MA0521-742RRR 34342041

[169] Ulges A Witsch EJ Pramanik G Klein M Birkner K Bühler U. Protein kinase CK2 governs the molecular decision between encephalitogenic T _H_ 17 cell and T _reg_ cell development. Proc Natl Acad Sci (2016) 113(36):10145–50. doi: 10.1073/pnas.1523869113 PMC5018788 27555590

[170] Gibson SA Yang W Yan Z Liu Y Rowse AL Weinmann AS. Protein kinase CK2 controls the fate between th17 cell and regulatory T cell differentiation. J Immunol (2017) 198(11):4244–54. doi: 10.4049/jimmunol.1601912 PMC5512439 28468969

[171] Gomez-Rodriguez J Wohlfert EA Handon R Meylan F Wu JZ Anderson SM. Itk-mediated integration of T cell receptor and cytokine signaling regulates the balance between Th17 and regulatory T cells. J Exp Med (2014) 211(3):529–43. doi: 10.1084/jem.20131459 PMC3949578 24534190

[172] Cho H-S Shin HM Haberstock-Debic H Xing Y Owens TD Funk JO. A small molecule inhibitor of ITK and RLK impairs th1 differentiation and prevents colitis disease progression. J Immunol (2015) 195(10):4822–31. doi: 10.4049/jimmunol.1501828 PMC4635571 26466958

[173] Gomez-Rodriguez J Sahu N Handon R Davidson TS Anderson SM Kirby MR. Differential expression of interleukin-17A and -17F is coupled to T cell receptor signaling *via* inducible T cell kinase. Immunity (2009) 31(4):587–97. doi: 10.1016/j.immuni.2009.07.009 PMC2767186 19818650

[174] Park Y Jin H-S Lopez J Elly C Kim G Murai M. TSC1 regulates the balance between effector and regulatory T cells. J Clin Invest (2013) 123(12):5165–78. doi: 10.1172/JCI69751 PMC3859395 24270422

[175] Shi LZ Wang R Huang G Vogel P Neale G Green DR. HIF1α–dependent glycolytic pathway orchestrates a metabolic checkpoint for the differentiation of TH17 and treg cells. J Exp Med (2011) 208(7):1367–76. doi: 10.1084/jem.20110278 PMC3135370 21708926

[176] Berod L Friedrich C Nandan A Freitag J Hagemann S Harmrolfs K. *De novo* fatty acid synthesis controls the fate between regulatory t and t helper 17 cells. Nat Med (2014) 20(11):1327–33. doi: 10.1038/nm.3704 25282359

[177] Matthias J Heink S Picard F Zeiträg J Kolz A Chao Y-Y. Salt generates antiinflammatory Th17 cells but amplifies pathogenicity in proinflammatory cytokine microenvironments. J Clin Invest (2020) 130(9):4587–600. doi: 10.1172/JCI137786 PMC7456214 32484796

[178] Arpaia N Campbell C Fan X Dikiy S van der Veeken J deRoos P. Metabolites produced by commensal bacteria promote peripheral regulatory T-cell generation. Nature (2013) 504(7480):451–5. doi: 10.1038/nature12726 PMC3869884 24226773

[179] Furusawa Y Obata Y Fukuda S Endo TA Nakato G Takahashi D. Commensal microbe-derived butyrate induces the differentiation of colonic regulatory T cells. Nature (2013) 504(7480):446–50. doi: 10.1038/nature12721 24226770

[180] Smith PM Howitt MR Panikov N Michaud M Gallini CA Bohlooly-Y M. The microbial metabolites, short-chain fatty acids, regulate colonic treg cell homeostasis. Science (2013) 341(6145):569–73. doi: 10.1126/science.1241165 PMC3807819 23828891

[181] Park J Kim M Kang SG Jannasch AH Cooper B Patterson J. Short-chain fatty acids induce both effector and regulatory T cells by suppression of histone deacetylases and regulation of the mTOR–S6K pathway. Mucosal Immunol (2015) 8(1):80–93. doi: 10.1038/mi.2014.44 24917457 PMC4263689

[182] Chen L Sun M Wu W Yang W Huang X Xiao Y. Microbiota metabolite butyrate differentially regulates th1 and th17 cells’ Differentiation and function in induction of colitis. Inflammatory Bowel Dis (2019) 25(9):1450–61. doi: 10.1093/ibd/izz046 PMC6701512 30918945

[183] Hui W Yu D Cao Z Zhao X. Butyrate inhibit collagen-induced arthritis *via* Treg/IL-10/Th17 axis. Int Immunopharmacol (2019) 68:226–33. doi: 10.1016/j.intimp.2019.01.018 30660077

[184] Zhang Y Li F Chen C Li Y Xie W Huang D. RAGE-mediated T cell metabolic reprogramming shapes T cell inflammatory response after stroke. J Cereb Blood Flow Metab (2022) 42(6):952–65. doi: 10.1177/0271678X211067133 PMC9125488 34910890

[185] Elahi A Sabui S Narasappa NN Agrawal S Lambrecht NW Agrawal A. Biotin Deficiency Induces Th1- and Th17-Mediated Proinflammatory Responses in Human CD4+ T Lymphocytes *via* Activation of the mTOR Signaling Pathway. J Immunol (2018) 200(8):2563–70. doi: 10.4049/jimmunol.1701200 PMC5893381 29531163

[186] Lopez Krol A Nehring HP Krause FF Wempe A Raifer H Nist A. Lactate induces metabolic and epigenetic reprogramming of pro-inflammatory Th17 cells. EMBO Rep (2022) 23(12):1–11. doi: 10.15252/embr.202254685 PMC9724659 36215678

[187] Koch MA Thomas KR Perdue NR Smigiel KS Srivastava S Campbell DJ. T-bet+ Treg cells undergo abortive th1 cell differentiation due to impaired expression of IL-12 receptor β2. Immunity (2012) 37(3):501–10. doi: 10.1016/j.immuni.2012.05.031 PMC3501343 22960221

[188] Santegoets SJ Duurland CL Jordanova ES van Ham JJ Ehsan I van Egmond SL. Tbet-positive regulatory T cells accumulate in oropharyngeal cancers with ongoing tumor-specific type 1 T cell responses. J ImmunoTherapy Cancer (2019) 7(1):14. doi: 10.1186/s40425-019-0497-0 PMC6339415 30658697

[189] Wohlfert EA Grainger JR Bouladoux N Konkel JE Oldenhove G Ribeiro CH. GATA3 controls Foxp3+ regulatory T cell fate during inflammation in mice. J Clin Invest (2011) 121(11):4503–15. doi: 10.1172/JCI57456 PMC3204837 21965331

[190] Lee Y Awasthi A Yosef N Quintana FJ Xiao S Peters A. Induction and molecular signature of pathogenic TH17 cells. Nat Immunol (2012) 13(10):991–9. doi: 10.1038/ni.2416 PMC3459594 22961052

[191] Gaublomme JT Yosef N Lee Y Gertner RS Yang LV Wu C. Single-cell genomics unveils critical regulators of th17 cell pathogenicity. Cell (2015) 163(6):1400–12. doi: 10.1016/j.cell.2015.11.009 PMC4671824 26607794

[192] Chung Y Chang SH Martinez GJ Yang XO Nurieva R Kang HS. Critical regulation of early th17 cell differentiation by interleukin-1 signaling. Immunity (2009) 30(4):576–87. doi: 10.1016/j.immuni.2009.02.007 PMC2705871 19362022

[193] Ghoreschi K Laurence A Yang X-P Tato CM McGeachy MJ Konkel JE. Generation of pathogenic TH17 cells in the absence of TGF-β signalling. Nature (2010) 467(7318):967–71. doi: 10.1038/nature09447 PMC3108066 20962846

[194] Hirota K Duarte JH Veldhoen M Hornsby E Li Y Cua DJ. Fate mapping of IL-17-producing T cells in inflammatory responses. Nat Immunol (2011) 12(3):255–63. doi: 10.1038/ni.1993 PMC3040235 21278737

[195] Basdeo SA Cluxton D Sulaimani J Moran B Canavan M Orr C. Ex-th17 (Nonclassical th1) cells are functionally distinct from classical th1 and th17 cells and are not constrained by regulatory T cells. J Immunol (2017) 198(6):2249–59. doi: 10.4049/jimmunol.1600737 28167631

[196] Noster R Riedel R Mashreghi M-F Radbruch H Harms L Haftmann C. IL-17 and GM-CSF expression are antagonistically regulated by human T helper cells. Sci Trans Med (2014) 6(241):241ra80. doi: 10.1126/scitranslmed.3008706 24944195

[197] Ramstein J Broos CE Simpson LJ Ansel KM Sun SA Ho ME. IFN-γ–producing T-helper 17.1 cells are increased in sarcoidosis and are more prevalent than T-helper type 1 cells. Am J Respir Crit Care Med (2016) 193(11):1281–91. doi: 10.1164/rccm.201507-1499OC PMC4910899 26649486

[198] Arger NK Machiraju S Allen IE Woodruff PG Koth LL. T-bet expression in peripheral th17.0 cells is associated with pulmonary function changes in sarcoidosis. Front Immunol (2020) 11:1129. doi: 10.3389/fimmu.2020.01129 32774332 PMC7387715

[199] Okada S Markle JG Deenick EK Mele F Averbuch D Lagos M. Impairment of immunity to Candida and Mycobacterium in humans with bi-allelic RORC mutations. Science (2015) 349(6248):606–13. doi: 10.1126/science.aaa4282 PMC4668938 26160376

[200] Sallusto F Cassotta A Hoces D Foglierini M Lanzavecchia A. Do memory CD4 T cells keep their cell-type programming: plasticity versus fate commitment? Cold Spring Harbor Perspect Biol (2018) 10(3):a029421. doi: 10.1101/cshperspect.a029421 PMC5830897 28432133

[201] Wang Y-H Voo KS Liu B Chen C-Y Uygungil B Spoede W. A novel subset of CD4+ TH2 memory/effector cells that produce inflammatory IL-17 cytokine and promote the exacerbation of chronic allergic asthma. J Exp Med (2010) 207(11):2479–91. doi: 10.1084/jem.20101376 PMC2964570 20921287

[202] Cosmi L Liotta F Maggi E Romagnani S Annunziato F. Th17 cells: new players in asthma pathogenesis. Allergy (2011) 66(8):989–98. doi: 10.1111/j.1398-9995.2011.02576.x 21375540

[203] McCluskey D Benzian-Olsson N Mahil SK Hassi NK Wohnhaas CT Burden AD. Single-cell analysis implicates TH17-to-TH2 cell plasticity in the pathogenesis of palmoplantar pustulosis. J Allergy Clin Immunol (2022) 150(4):882–93. doi: 10.1016/j.jaci.2022.04.027 35568077

[204] Morita R Schmitt N Bentebibel S-E Ranganathan R Bourdery L Zurawski G. Human blood CXCR5+CD4+ T cells are counterparts of T follicular cells and contain specific subsets that differentially support antibody secretion. Immunity (2011) 34(1):108–21. doi: 10.1016/j.immuni.2010.12.012 PMC3046815 21215658

[205] Ma CS Wong N Rao G Avery DT Torpy J Hambridge T. Monogenic mutations differentially affect the quantity and quality of T follicular helper cells in patients with human primary immunodeficiencies. J Allergy Clin Immunol (2015) 136(4):993–1006.e1. doi: 10.1016/j.jaci.2015.05.036 26162572 PMC5042203

